# Postoperative Hirschsprung’s associated enterocolitis (HAEC): transition zone as putative histopathological predictive factor

**DOI:** 10.1136/jcp-2023-209129

**Published:** 2023-11-23

**Authors:** Miriam Duci, Luisa Santoro, Angelo Paolo Dei Tos, Greta Loss, Claudia Mescoli, Piergiorgio Gamba, Francesco Fascetti Leon

**Affiliations:** 1 Pediatric Surgery Unit, Department of Women's and Children's Health, Università degli Studi di Padova, Padova, Italy; 2 Surgical Pathology and Cytopathology Unit, Department of Medicine - DIMED, University of Padova School of Medicine, Padova, Italy

**Keywords:** Gastrointestinal Diseases, Histology, INFLAMMATION

## Abstract

**Aims:**

Hirschsprung’s-associated enterocolitis (HAEC) is the most severe complication of Hirschsprung disease (HD), and its pathogenesis is still unknown. Length of transition zone (TZ) interposed between aganglionic and normal bowel has been poorly explored as predictor for postoperative HAEC (post-HAEC). This study aimed to identify potential predictive factors for post-HAEC, with a particular focus on histopathological findings.

**Methods:**

Data from Hirschsprung patients treated in a single Italian centre between 2010 and 2022 with a follow-up >6 months were collected. Thorough histopathological examination of the resected bowel was conducted, focusing on length of TZ and aganglionic bowel.The degree of inflammatory changes in ganglionic resected bowel was further obtained. Ultra-long HD, total colonic aganglionosis and ultra-short HD were excluded. Bivariate and multivariate regression analysis were performed.

**Results:**

Thirty-one patients were included; 5 experienced preoperative HAEC (pre-HAEC) and later post-HAEC (16.1%), further 10 patients developed post-HAEC (total post-HAEC 48.38%). Pre-HAEC-history and a TZ<2.25 cm correlated with an early development of post-HAEC. Multivariate analysis identified a TZ<2.25 cm as an independent post-HAEC predictive factor (p=0.0096). Inflammation within the ganglionic zone and a TZ<2.25 cm correlated with higher risk of post-HAEC (p=0.0074, 0.001, respectively). Severe post-HAEC more frequently occurred in patients with pre-HAEC (p=0.011), histological inflammation (p=0.0009) and short TZ (p=0.0015).

**Conclusions:**

This study suggests that TZ<2.25 cm predicts the risk of post-HAEC. Preoperative clinical and histopathology inflammation may predispose to worst post-HAEC. Readily available histopathological findings might help identifying patients at higher risk for HAEC and implementing prevention strategies.

**WHAT IS ALREADY KNOWN ON THIS TOPIC**

**WHAT THIS STUDY ADDS**

- This study reveals that a transition zone (TZ)<2.25 cm independently predicts the risk of post-HAEC. Additionally, an inflamed proximal segment of the resected colon, pre-HAEC history and a short TZ are correlated with a more severe grade of post-HAEC. An earlier onset of post-HAEC appears to be associated with a pre-HAEC history and a TZ<2.25 cm.

**HOW THIS STUDY MIGHT AFFECT RESEARCH, PRACTICE OR POLICY**

- These findings suggest that paediatric surgeons should counsel the parents of children with HD about the potential risks of worse outcomes related to the extension of TZ. It is also important be alert when encountering children with an inflamed proximal segment of the resected colon, because of their increased risk of subsequently developing early and severe postoperative HAEC. Based on these results, we recommend routine examination of resected bowel tissue from children with HD for precise measurement of the TZ length and assessment of inflammatory mucosal grade.

## Introduction

 Hirschsprung disease (HD) is a congenital disease characterised by the absence of ganglionic cells in the distal bowel, extending proximally for varying distances. A variety of diagnostic tests including contrast enema and anorectal manometry may be used as diagnostic screens, but diagnosis ultimately laysupon histopathological examination of a rectal biopsy.^1^ The diagnostic histological features of HD include the absence of ganglion cells and an increase in hypertrophic cholinergic nerves. However, immunohistochemistry may be necessary to facilitate the diagnosis. The most widely applied ancillary methods are acetylcholinesterase (AChE) and calretinin stainings. An increased expression of AChE in hypertrophic nerve fibres and the complete absence of calretinin-positive mucosal nerves indicate aganglionic rectal tissue.^2^ Once the diagnosis has been made, the treatment of this disease involves resecting the affected colon, based on intraoperative biopsies. Resection extends to variable length of normal bowel to prevent relapse due to unrecognised diseased remnant. In fact, edges between normally innervated bowel and affected tissue can be irregular along the bowel circumference. The most severe and potentially life-threatening complication of HD is Hirschsprung’s associated enterocolitis (HAEC), which can develop either preoperatively (in up to 30%) or after a surgical correction (in up to 50% of patients).^3^ Its associated mortality rate has decreased significantly from 30% in the 1980s to around 1% in the 2010s. Early recognition and management of HAEC’s signs and symptoms are crucial for successful treatment.^4^,^8^ Nevertheless, the pathogenesis of HAEC remains poorly understood involving different mechanisms such as intestinal epithelial barrier disruption, dysfunctional enteric innervation, bacterial translocation and altered microbiome. The length of the HD-affected region and the preoperative HAEC are the most mentioned prognostic risk factors. However, the reported data on these factors are not consistent in the current literature.^9^,^11^ Understanding the histopathological changes associated with the risk of developing postoperative-HAEC may provide insight into its pathophysiology and may help clinicians to implement preventive strategies. Therefore, this study aims to identify predictive factors associated with the development of postoperative-HAEC, focusing on pathological findings such as length of segments with altered neuroenteric system, and mucosal inflammatory changes in resected bowel.

## Materials and methods

This study is based on the pathological review of specimens collected during surgical treatment of HD cases consecutively diagnosed during the period from January 2010 to January 2022 in a single Italian referral Centre. A diagnosis was based on the absence of ganglion cells from either a suction rectal biopsy or an open rectal strip biopsy prior to the surgical correction. All H&E slides were examined by conventional light microscopy by two author investigators, each of whom worked independently and were blinded to the disease phenotype (ie, short-segment vs long-segment disease). Cases of ultra-long HD, total colonic aganglionosis and ultra-short HD were deliberately excluded from the study. All patients underwent a laparoscopic-assisted pull-through and further frozen sections analysis of the proximal margin of the pull-trough specimen was performed intraoperatively to confirm normal innervation of the pulled-through bowel as currently recommended.^8 12 13^ The resected specimens were embedded in paraffin and orientated in the sagittal plane. The review protocol for the study aimed to the length of the aganglionic and transition zone (TZ) and to the grade of mucosal inflammation within the resected part of the colon with normal representation of ganglion cells. In parallel, patient’s characteristics were collected from clinical records with particular focus on pre-HAEC and post-HAEC.

### Zones lengths and inflammatory changes

**Figure 1:**
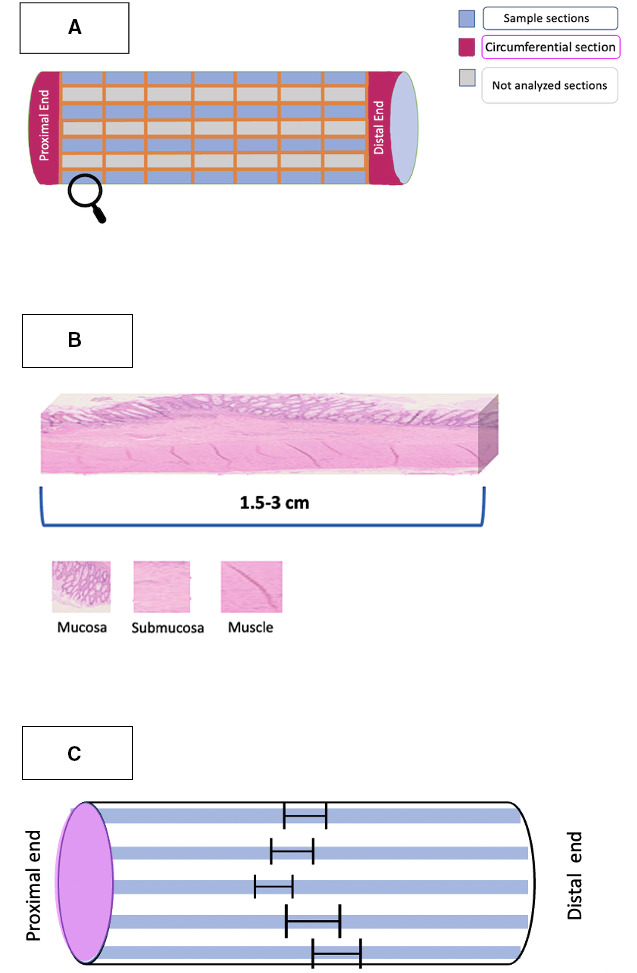
(A) Sampling method of the resected specimen: transverse full-circumference sections was performed at the proximal and the distal end (fuchsia sections). Multiple longitudinal sections were obtained to demonstrate a transitional point between aganglionic and ganglionic zone, defining the exact length of transition zone (blue sections). The cutting points were showed in the figure as orange lines. (B) Detail of sampled segment: microscopic examination was used to evaluate clearly the distribution of ganglionic cells and hypertrophic nerves in order to measure the different zones. (C) The map of the TZ’S length: The map of the zones representation in the specimen was constructed based on the lengths of each single segment (black marker). As shown in the figure, in case of irregular profile of the zone’s edges, the lengths were calculated as a mean of the single value. TZ, transition zone.

The aganglionic zone was defined by the absence of ganglionic cells at the myenteric and submucosa layer. Hypertrophic nerves greater than 40 microns in diameter, located in the submucosa and myenteric plexuses were considered adjunctive criterium to define aganglionic zone.^14^ The TZ was determined based on previously published criteria by Kapur and Kennedy.^14 15^ Specifically, it was defined by one or more of the following neuropathological features: partial circumferential aganglionosis (absence myenteric and/or submucosal ganglion cells in a contiguous eight of the circumference), myenteric hypoganglionosis (as defined above >1/8th circumference) or submucosal nerve hypertrophy (eg, >2 submucosal nerves >40 µm thick in one high power field) in the tract proximal to the aganglionic zone. The normo-ganglionic bowel was the contiguous segment, located cranially to the TZ, where none of the histological features of either the TZ or the aganglionic zone were seen. The map of the zones representation in the specimen was constructed from multiple longitudinal sections sampled in a defined scheme as shown in figure 1A,B. The sampling method allowed the measurement of the total extent of the histological zones. In cases where the edges of the zones exhibited irregular profiles, we implemented a standardised approach for measurements (figure 1C). The lengths of these zones were calculated as the mean of the values, maintaining consistency and precision in our measurements. This meticulous method of measurement was designed to provide a comprehensive and accurate assessment of the TZ. All measure were conducted after the formalin fixation process in accordance with the standardised centre protocol. Histological grading of inflammation in the ganglionic and TZ was assessed and graded from 0 to 5 according to previously published criteria by Teitelbaum *et al*^16^ (online supplemental file 1).

### Clinical data collection

The following data were retrospectively collected from patients: family history of HD, gestational age, birth weight, gender, age, associated syndromes, onset of signs and symptoms, preoperative HAEC and its severity defined according to Gosain *et al*.^17^ Further data collected concerning HD treatment and management, including the type and timing of the surgical procedure, the leucocytes counts and C reactive protein (CRP) level after at least 5 days of surgery were collected. The outcome parameters included the post-HAEC development, its severity, its timing after surgery and the associated mortality. Medical history positive for HAEC were defined according to Pastor criteria.^18^Patients were treated with oral or parenteral antibiotics based on HAEC severity. Severe HAEC was considered with at least a grade II according to Gosain score.^17^ A minimum follow-up of 6 months was considered.

### Statistical analysis

For categorical variables, Fisher’s exact test was used. For continuous variables, The Wilcoxon test or Kruskal-Wallis was applied. A Kaplan-Meier analysis was used to assess the risk factors associated with the time to first postoperative HAEC (post-HAEC). Proportional hazards regression modelling was performed for multivariate analysis to identify independent predictors of post-HAEC development. Statistical analysis was performed using the Statistical Package SAS V.9.4 (SAS Institute). Two-sided p values less than 0.05 were considered statistically significant.

## Results

During the study period, a total of 31 eligible patients were identified. Table 1 shows the demographic characteristics of the entire study population. The diagnosis of HD was confirmed histologically by rectal biopsies in all patients. Among them, 27 children (87%) underwent a primary pull-through procedure, while 4 children underwent a staged procedure with initial level-colostomy before the pull-through. Intraoperatively, aganglionic tract was identified up to the rectosigmoid junction in 22 patients (70%), up to sigmoid-descending colon in 5 patients (17%), up to splenic flexure in 3 (9.7%), and up to hepatic flexure in 1 (3.3%). Preoperative HAEC (pre-HAEC) was found in five patients (16.1%): three developed pre-HAEC of grade II and two of grade III. All patients who developed pre-HAEC also experienced post-HAEC.A total of 15 patients developed post-HAEC (48.38%) at the median onset of 90 days (range 15–320) after surgery with 7 cases classified as grade I HAEC, 7 as grade II, and one as grade III. After pathology revision, multivariate regression analysis identified a TZ<2.25 cm as an independent predictive factor for post-HAEC development (p=0.0096) (table 2).

**Table 1:** Clinical characterisation of the population * 3 Trisomy 21; 1 Coffin-Sirin syndrome

Characteristics	HD patients
M/F—n (%)	28 (90)/3 (10)
Gestational age (week)—median (range)Birth weight (g)—median (range)	38.55 (32–40.5)3400 (1115–4170)
Familiarity—n (%)	2 (6.45)
Associated anomalies—n (%)	4(12.9)

HDHirschsprung disease

**Table 2:** Independent predictors of post-HAEC or OR

Variables	Value	OR	95% CI	P value
Preoperative-HAEC	1 vs 0	1.630	0.513 to 5.180	0.475
TZ length (cm)	>2.25 vs <2.25	0.112	0.021 to 0.588	0.0096
Histological inflammation	1 vs 0	3.595	0.931 to 13.891	0.0635

HACEHirschsprung’s associated enterocolitisTZtransition zone

**Figure 2:**
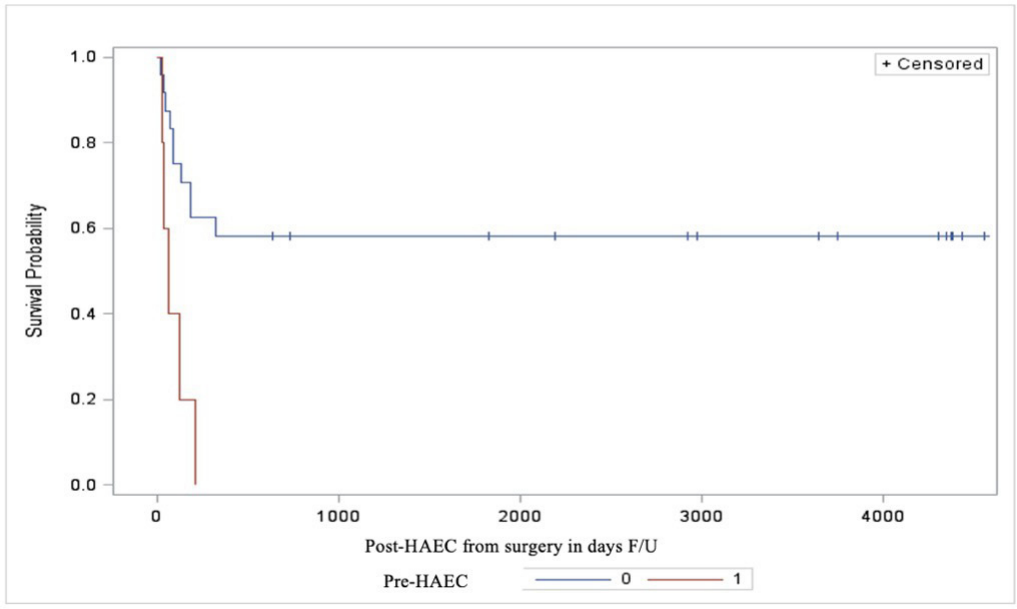
Kaplan-Mayer curves in two groups (pre-HAEC and no pre-HAEC) showing the time of developing post-HAEC in days after surgery. HAEC, Hirschsprung’s associated enterocolitis.

Bivariate analysis revealed that the inflammatory changes in the ganglionic resected bowel significantly correlated with the risk of post-HAEC (p=0.0074). Furthermore, severe post-HAEC occurred more frequently in patients with pre-HAEC (p=0.011), TZ<2.25 cm (p=0.0015) and the presence of histological inflammation, any grade (p=0.0009).The correlation between severity of histological inflammation and the severity of post-HAEC did not reach statistical significance (p=0.058) (table 3). Moreover, a history of pre-HAEC and a TZ<2.25 cm was associated with an early development of post-HAEC, as shown by Kaplan-Mayer curves (89.6 vs 116 days and 95 vs 162 days, respectively) (figures2  3). No significant differences were observed in terms of white cells counts and serum CRP level measured predischarge in post-HAEC group vs no-HAEC group (p=0.7 and p=0.110, respectively) (online supplemental file 2). The mortality related to HAEC was 3.2% and occurred in a patient with associated Down’s syndrome.

**Table 3:** Correlation between HAEC risk factors and severity of post-HA

	Grade post-HAEC			
	0 (N=16)	1 (N=7)	2+3 (N=8)	P value
TZ length (cm) median (range)	3.5 (1.5–5.3)	1.4 (0.5–4.8)	1.5 (0.4–3.1)	0.0009
Histological Inflammation n(%)	3 (21.4)	7 (100)	5 (62.5)	0.0015
Max Grade of inflammation median (range)	0 (0–5)	1 (1-3)	1.5 (0–4)	0.058
Preoperative-HAEC n (%)	0 (0)	1 (14.3)	4 (50)	0.011

HACEHirschsprung’s associated enterocolitisTZtransition zone

**Figure 3:**
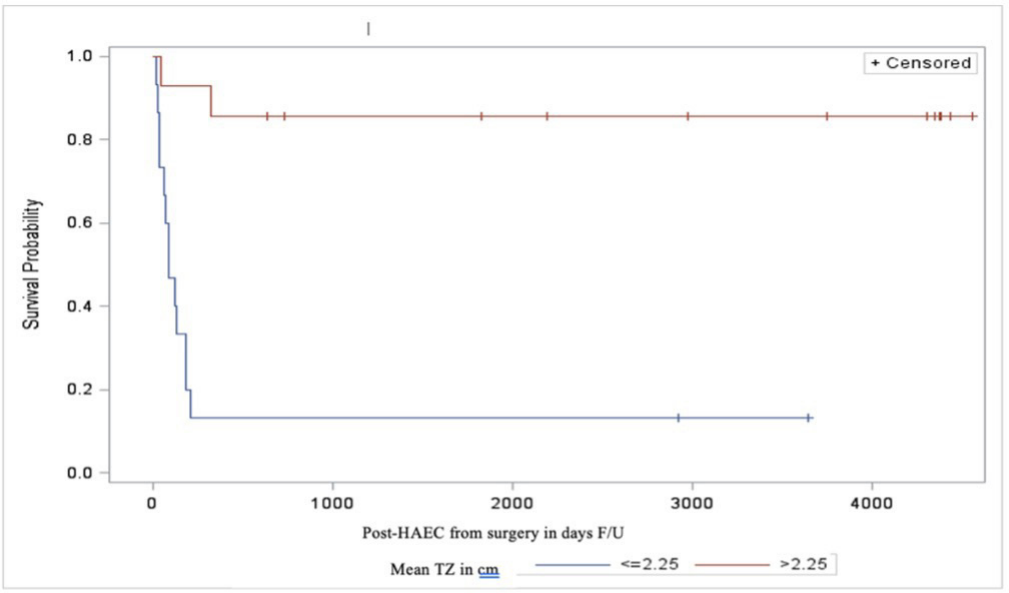
Kaplan-Mayer curves in two groups (TZ<2.25 and TZ>2.25) showing the time of developing post-HAEC in days after surgery. HAEC, Hirschsprung’s associated enterocolitis; TZ, transition zone.

## Discussion

HACE is a severe and potentially life-threatening condition, accounting for approximately half of the deaths associated with HD.^19 20^ The wide variation in reported incidence rates of post-HAEC (2%–42%) is most likely due to the lack of a standardised definition for HAEC.^4^,^8^ However, diagnosing patients with Pastor scores greater than four as having HAEC has been found to be a pplicable in clinical practice.^13 18 21^ In our study period, 48% of patients developed post-HAEC. This result aligns with the higher end of reported series, probably due to the awareness towards such entity in a referral centre.

While recent studies have mainly focused on identifying potential mechanisms involved in the etiopathogenesis of preoperative HAEC, few studies have explored the relationship between pathological findings, inflammatory status of the affected intestinal tract and the risk of postoperative enterocolitis.^45 7 10 22^,^24^

In particular, the results of studies on post-HAEC are conflicting. Some authors reported long aganglionic segments to be strongly associated with post-operative HAEC while others did not confirm this association. Consistent with these findings, the present study depicted that having a longer-segment HD increases the risk of postoperative HAEC. The exact reason why patients with longer aganglionic segments have a higher risk of developing HAEC remain incompletely understood, even though the abnormal development of enteric nervous system (ENS) is known to play an important role. In fact, the ENS is involved in almost all aspect of gastrointestinal function, including intestinal barrier and immune response to the intestinal microbiome.^25^ Patients with longer aganglionic tracts tend to produce the proximal bowel obstruction and generate more significant intraluminal pressure, leading to intestinal dysmotility and a higher vulnerability to bacteria stasis. A recent study speculated that patients with longer aganglionic segment exhibited a lower microbiota diversity and an overabundance of harmful microbial species, leading the intestine to be more susceptible to enteric inflammation compared with those with shorter aganglionic segment.^26^

The present study revealed for the first time that a short TZ independently predicts post-HAEC. In addition, patients with a TZ less than 2.25 cm are more prone to develop severe post-HAEC with earlier onset compared with those with a longer TZ. The length of the TZ, which can vary significantly, has never been explored as a risk factor of post-HAEC development. We speculate that this finding might be a consequence of an unbalance in intestinal homeostasis, including impaired epithelial barrier function, compromised mucosal immunity and dysregulation of the gut microbiome, which become particularly pronounced when the TZ is short. This would confer to the TZ a buffer role between different microbiome zones.

It is noteworthy that the variability on the clinical picture of HD patients is not fully explained by the length of aganglionic colon. Neuro Enteric System abnormalities, other than aganglionosis and hypertrophy of nerves, possibly expressed in the whole bowel of patients justify the diverse diseases pattern.^25 27^ Therefore, the length of the TZ may be one the possible markers of specific phenotypes.

Dysfunction of ganglionic cells may lead to an abnormal intestinal balance, in which some perturbations such as bacterial overgrowth or partial obstruction may cause inflammation and consequently enterocolitis. In fact, some studies reported that histopathological inflammation on the resected bowel correlated with a higher risk of developing post-HAEC.^5 11 28 29^ Consistent with previous studies, our data demonstrated that histological inflammation in the ganglionic area is associated with the occurrence of post-HAEC. However, when comparing the severity of histopathological grade to the HAEC score, our study did not confirm the positive correlation reported in one previous study.^30^This discrepancy could be explained by the uneven distribution of HAEC histological changes in different parts of the intestine, that in this study has not been fully explored. Regarding the examination of the proximal resection margin, we also meticulously explored the distribution and appearance of ganglion cells but did not identify any specific abnormalities that could account for the occurrence of HAEC.

Previous literature has suggested a significant increase in the incidence of postoperative HAEC in patients who experienced preoperative HAEC.^31^ In our study, pre-HAEC was not associated independently with post-HAEC, but it was associated with more severe and earlier occurrence of HAEC, a result also reported by Sakurai *et al*.^24^ As a possible explanation for these associations, it is likely that preoperative HAEC alter the protecting mechanisms in the intestine, making patients more susceptible to experience more severe and earlier HAEC episodes compared with those without a history of preoperative HAEC events. We investigated the possible correlation between systemic signs of inflammation No association between leucocyte count or CRP level and post-HAEC development were found. This may suggest that the pathogenesis of HAEC is not primarily related to systemic inflammation but rather to mucosal damage, which could have a more localised impact.

The present study is not without limitations, including the retrospective collection of clinical data in a single institution with a limited number of patients, that may have influenced the statistical power. However, our results may be the base for a thorough patients counselling including the potential risk of worse outcomes related to the extension of TZ. In addition, when encountering children with an inflamed proximal segment of the resected colon, clinicians should be alert because of their increased risk of subsequently developing early and severe postoperative HAEC. Furthermore, we suggest that resected bowel tissue from children with HD should be routinely examined for precise TZ length and inflammatory mucosal grade. Standardised methods in a multicentre setting is advisable for confirming this findings.

## supplementary material

10.1136/jcp-2023-209129 online supplemental file 1

10.1136/jcp-2023-209129 online supplemental file 2

## Data Availability

All data relevant to the study are included in the article or uploaded as online supplemental information.
