# Supplementary material for: Postoperative Hirschsprung’s associated enterocolitis (HAEC): transition zone as putative histopathological predictive factor
Source: J Clin Pathol. 2023 Nov 23;78(2):e209129. doi: 10.1136/jcp-2023-209129 (PMC11874427; doi:10.1136/jcp-2023-209129)
Supplement: online supplemental file 1 [file jcp-78-2-s001.pdf]

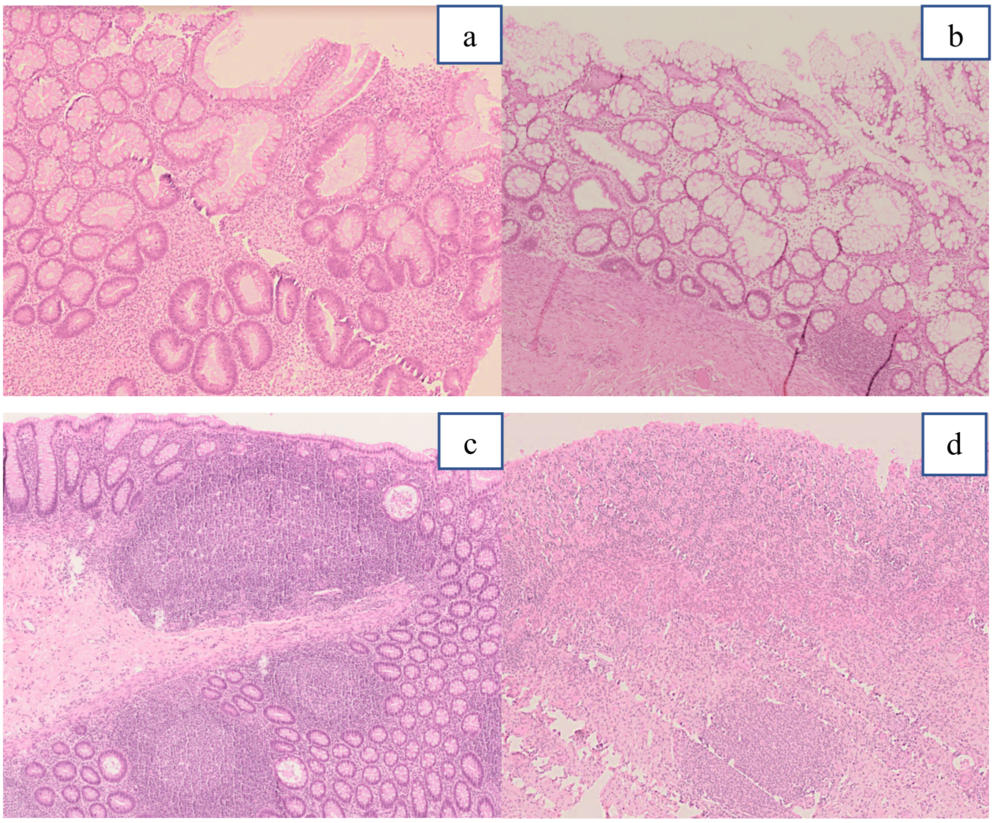

Supplementary file 1*: Histopathological grades of HAEC based on Teitelbaun classification (5X magnification).*[16] *Grade 1 shows shows crypt dilation associated to mucin retention (a); grade II is characterized by cryptitis (multiple crypt dilation and mucin retention) (b); grade III is depicted by different crypt abscesses (c); grade IV describes mucosal ulceration (d); grade V shows transmural necrosis or perforation (not shown)*
