# Supplementary material for: Postoperative Hirschsprung’s associated enterocolitis (HAEC): transition zone as putative histopathological predictive factor
Source: J Clin Pathol. 2023 Nov 23;78(2):e209129. doi: 10.1136/jcp-2023-209129 (PMC11874427; doi:10.1136/jcp-2023-209129)
Supplement: online supplemental file 2 [file jcp-78-2-s002.pdf]

Variables	Post-HAEC	no Post-HAEC	p-value
**CRP mg/l- median(range)**	5.3(2-11.5)	9.8 (0-37.2)	0.110
**WBC n/mmc- median (range)**	10.83 (4.81-17.68)	9.8 (6.1 -13)	0.70

Supplementary file2: Laboratory values measured pre-discharge.
